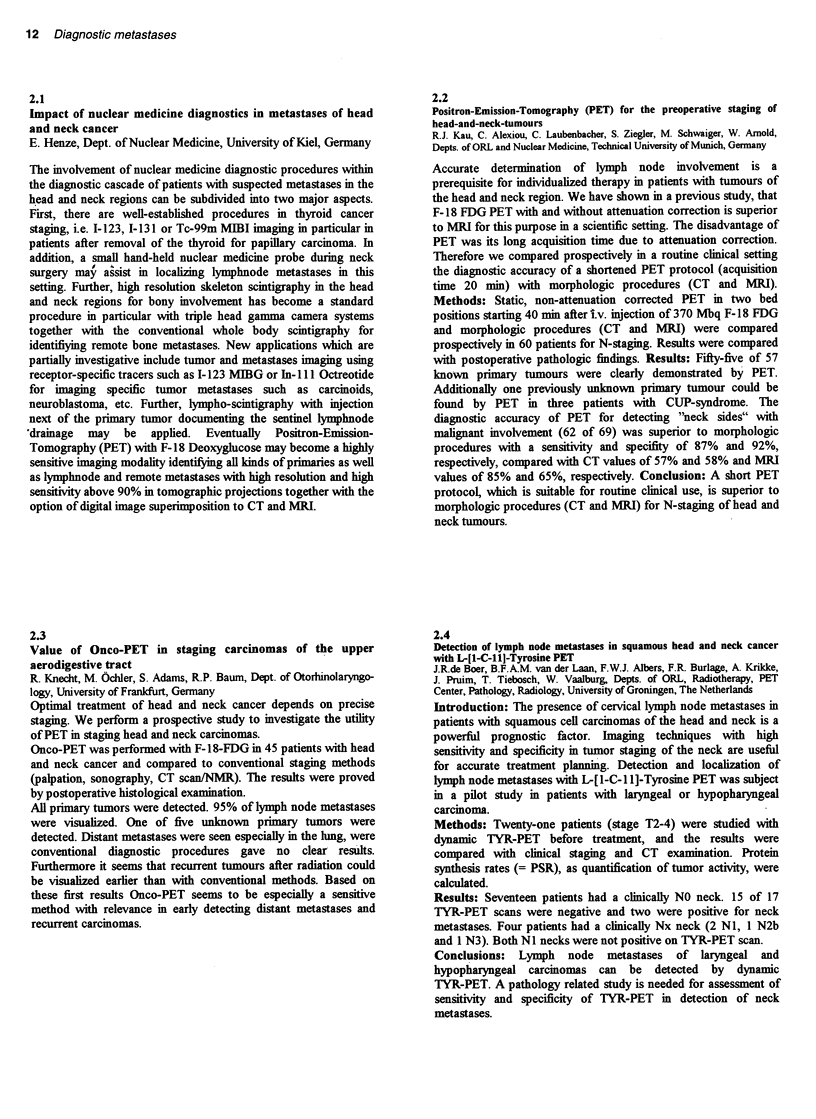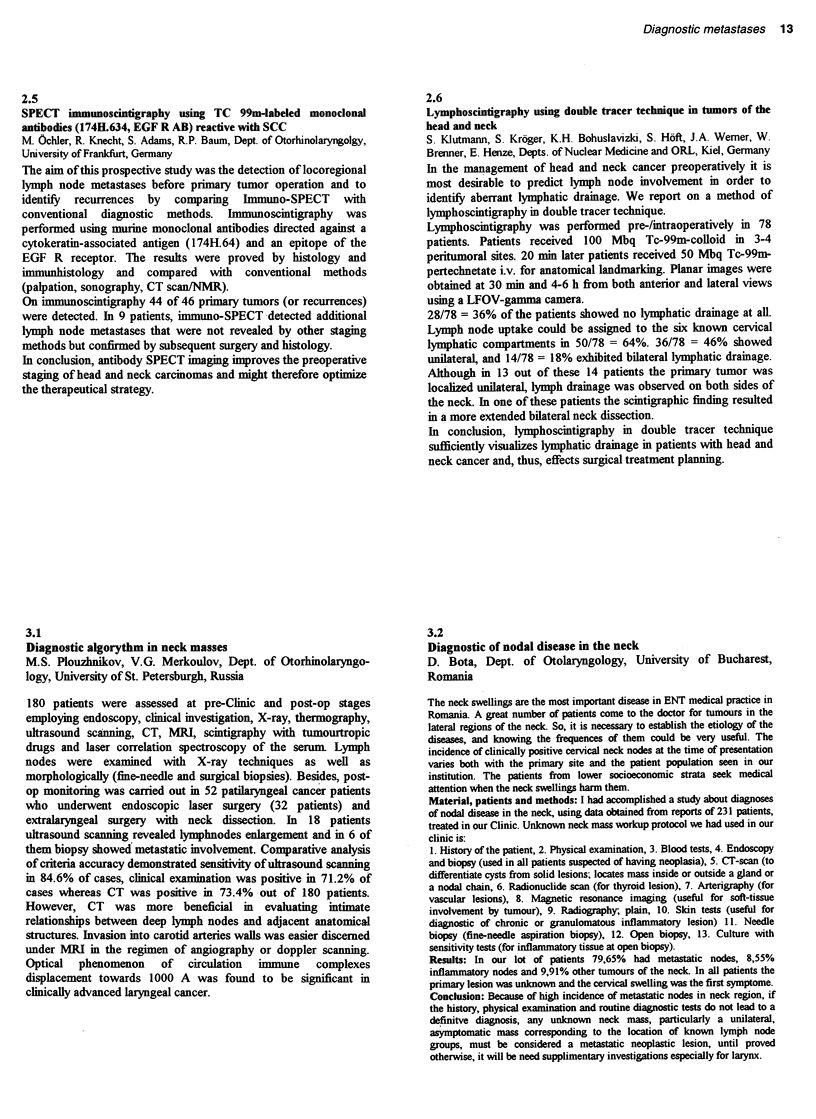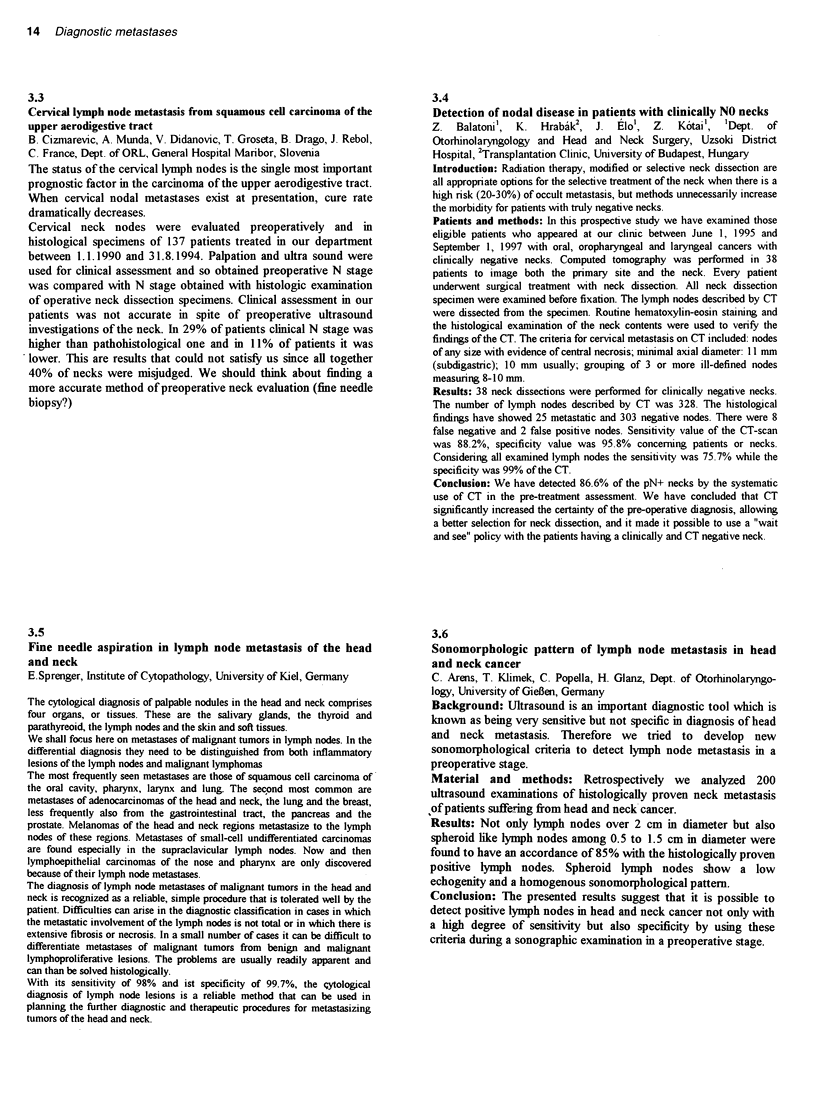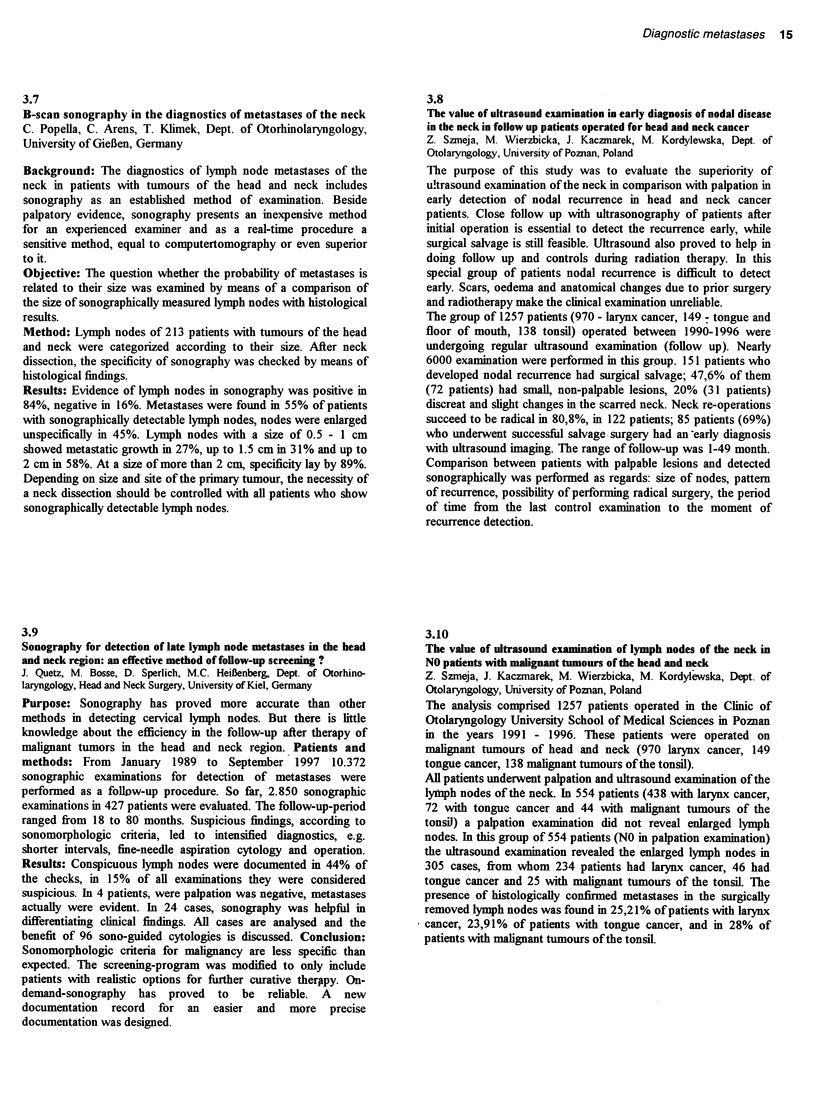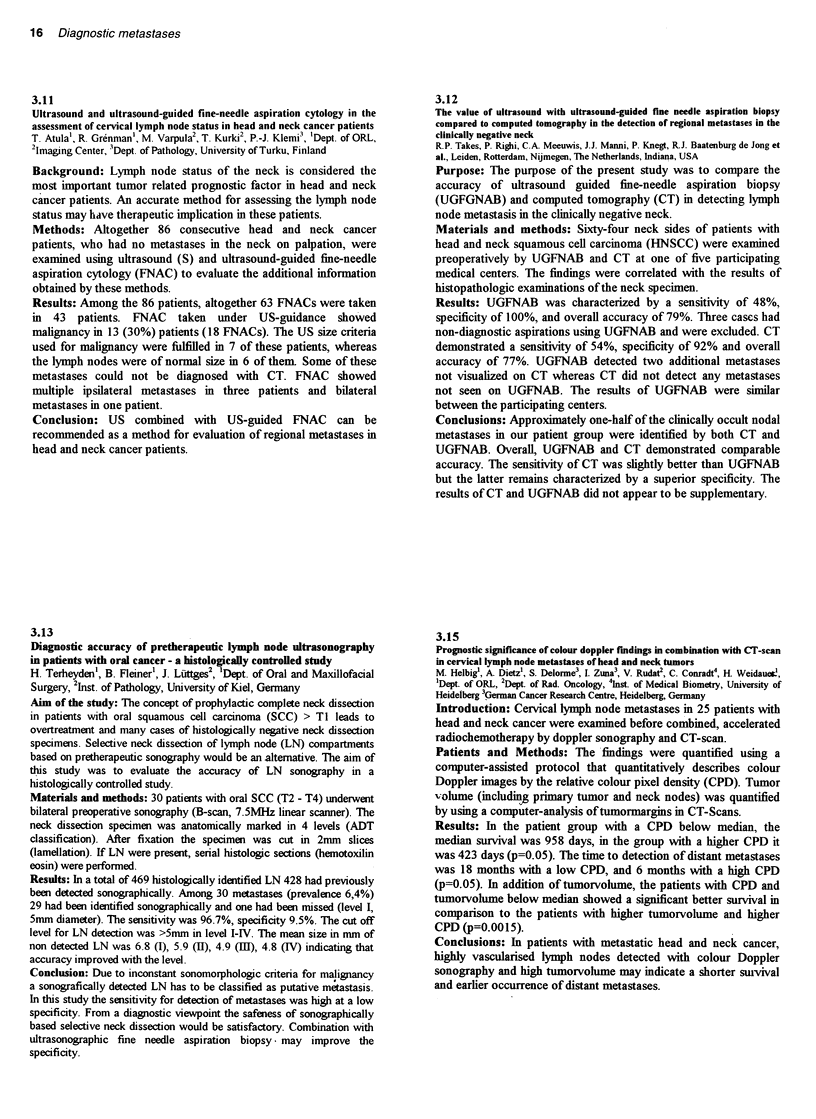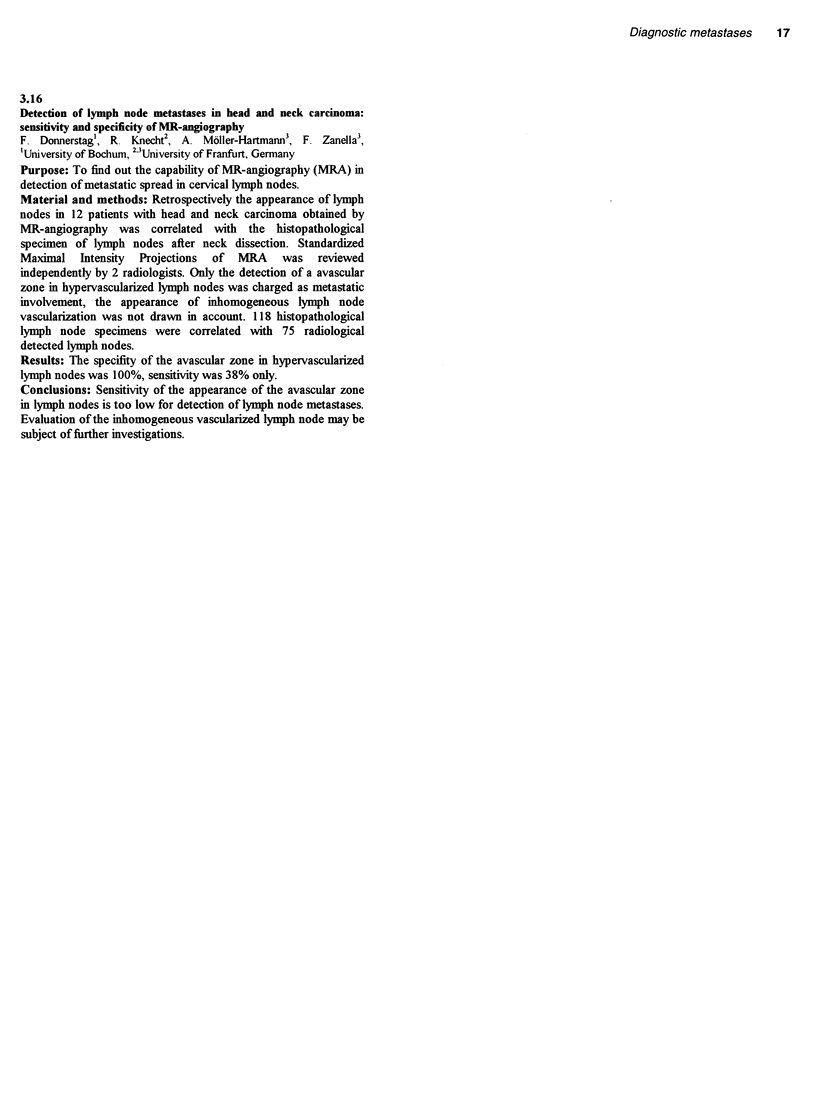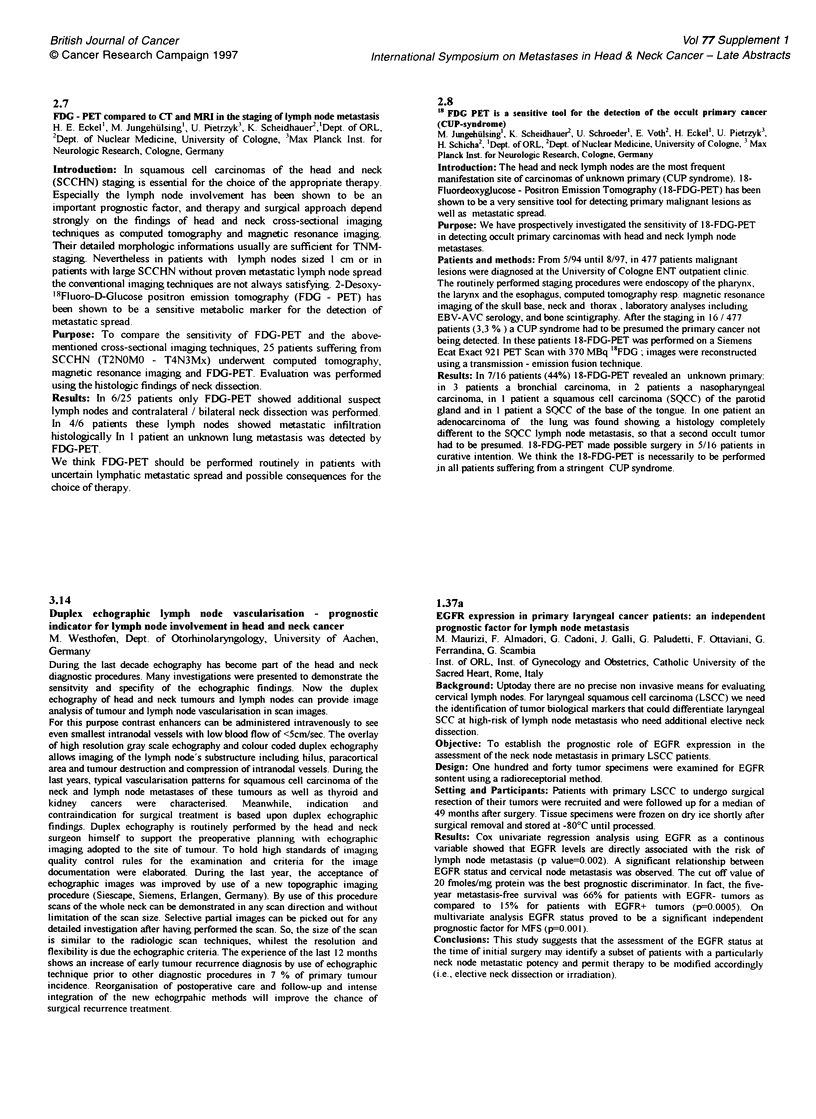# Diagnosis of Metastases

**Published:** 1998

**Authors:** 


### 2.1
### Impact of nuclear medicine diagnostics in metastases of head and neck cancer
E. Henze, Dept. of Nuclear Medicine, University of Kiel, Germany

The involvement of nuclear medicine diagnostic procedures within the diagnostic cascade of patients with suspected metastases in the head and neck regions can be subdivided into two major aspects. First, there are well-established procedures in thyroid cancer staging, i.e. I-123, I-131 or Tc-99m MIBI imaging in particular in patients after removal of the thyroid for papillary carcinoma. In addition, a small hand-held nuclear medicine probe during neck surgery may assist in localizing lymphnode metastases in this setting. Further, high resolution skeleton scintigraphy in the head and neck regions for bony involvement has become a standard procedure in particular with triple head gamma camera systems together with the conventional whole body scintigraphy for identifiying remote bone metastases. New applications which are partially investigative include tumor and metastases imaging using receptor-specific tracers such as I-123 MIBG or In-111 Octreotide for imaging specific tumor metastases such as carcinoids, neuroblastoma, etc. Further, lympho-scintigraphy with injection next of the primary tumor documenting the sentinel lymphnode drainage may be applied. Eventually Positron-Emission-Tomography (PET) with F-18 Deoxyglucose may become a highly sensitive imaging modality identifying all kinds of primaries as well as lymphnode and remote metastases with high resolution and high sensitivity above 90% in tomographic projections together with the option of digital image superimposition to CT and MRI.

### 2.2
### Positron-Emission-Tomography (PET) for the preoperative staging of head-and-neck-tumours
R.J. Kau, C. Alexiou, C. Laubenbacher, S. Ziegler, M. Schwaiger, W. Arnold, Depts. of ORL and Nuclear Medicine, Technical University of Munich, Germany

Accurate determination of lymph node involvement is a prerequisite for individualized therapy in patients with tumours of the head and neck region. We have shown in a previous study, that F-18 FDG PET with and without attenuation correction is superior to MRI for this purpose in a scientific setting. The disadvantage of PET was its long acquisition time due to attenuation correction. Therefore we compared prospectively in a routine clinical setting the diagnostic accuracy of a shortened PET protocol (acquisition time 20 min) with morphologic procedures (CT and MRI). **Methods:** Static, non-attenuation corrected PET in two bed positions starting 40 min after i.v. injection of 370 Mbq F-18 FDG and morphologic procedures (CT and MRI) were compared prospectively in 60 patients for N-staging. Results were compared with postoperative pathologic findings. **Results:** Fifty-five of 57 known primary tumours were clearly demonstrated by PET. Additionally one previously unknown primary tumour could be found by PET in three patients with CUP-syndrome. The diagnostic accuracy of PET for detecting "neck sides" with malignant involvement (62 of 69) was superior to morphologic procedures with a sensitivity and specifity of 87% and 92%, respectively, compared with CT values of 57% and 58% and MRI values of 85% and 65%, respectively. **Conclusion:** A short PET protocol, which is suitable for routine clinical use, is superior to morphologic procedures (CT and MRI) for N-staging of head and neck tumours.

### 2.3
### Value of Onco-PET in staging carcinomas of the upper aerodigestive tract
R. Knecht, M. Öchler, S. Adams, R.P. Baum, Dept. of Otorhinolaryngology, University of Frankfurt, Germany

Optimal treatment of head and neck cancer depends on precise staging. We perform a prospective study to investigate the utility of PET in staging head and neck carcinomas.

Onco-PET was performed with F-18-FDG in 45 patients with head and neck cancer and compared to conventional staging methods (palpation, sonography, CT scan/NMR). The results were proved by postoperative histological examination.

All primary tumors were detected. 95% of lymph node metastases were visualized. One of five unknown primary tumors were detected. Distant metastases were seen especially in the lung, were conventional diagnostic procedures gave no clear results. Furthermore it seems that recurrent tumours after radiation could be visualized earlier than with conventional methods. Based on these first results Onco-PET seems to be especially a sensitive method with relevance in early detecting distant metastases and recurrent carcinomas.

### 2.4
### Detection of lymph node metastases in squamous head and neck cancer with L-[1-C-11]-Tyrosine PET
J.R.de Boer, B.F.A.M. van der Laan, F.W.J. Albers, F.R. Burlage, A. Krikke, J. Pruim, T. Tiebosch, W. Vaalburg, Depts. of ORL, Radiotherapy, PET Center, Pathology, Radiology, University of Groningen, The Netherlands

**Introduction:** The presence of cervical lymph node metastases in patients with squamous cell carcinomas of the head and neck is a powerful prognostic factor. Imaging techniques with high sensitivity and specificity in tumor staging of the neck are useful for accurate treatment planning. Detection and localization of lymph node metastases with L-[1-C-11]-Tyrosine PET was subject in a pilot study in patients with laryngeal or hypopharyngeal carcinoma.

**Methods:** Twenty-one patients (stage T2-4) were studied with dynamic TYR-PET before treatment, and the results were compared with clinical staging and CT examination. Protein synthesis rates (= PSR), as quantification of tumor activity, were calculated.

**Results:** Seventeen patients had a clinically N0 neck. 15 of 17 TYR-PET scans were negative and two were positive for neck metastases. Four patients had a clinically Nx neck (2 N1, 1 N2b and 1 N3). Both N1 necks were not positive on TYR-PET scan.

**Conclusions:** Lymph node metastases of laryngeal and hypopharyngeal carcinomas can be detected by dynamic TYR-PET. A pathology related study is needed for assessment of sensitivity and specificity of TYR-PET in detection of neck metastases.

### 2.5
**SPECT immunoscintigraphy using TC 99m-labeled monoclonal antibodies (174H.634, EGF R AB) reactive with SCC**

M. Öchler, R. Knecht, S. Adams, R.P. Baum, Dept. of Otorhinolaryngolgy, University of Frankfurt, Germany

The aim of this prospective study was the detection of locoregional lymph node metastases before primary tumor operation and to identify recurrences by comparing Immuno-SPECT with conventional diagnostic methods. Immunoscintigraphy was performed using murine monoclonal antibodies directed against a cytokeratin-associated antigen (174H.64) and an epitope of the EGF R receptor. The results were proved by histology and immunhistology and compared with conventional methods (palpation, sonography, CT scan/NMR).
On immunoscintigraphy 44 of 46 primary tumors (or recurrences) were detected. In 9 patients, immuno-SPECT detected additional lymph node metastases that were not revealed by other staging methods but confirmed by subsequent surgery and histology.
In conclusion, antibody SPECT imaging improves the preoperative staging of head and neck carcinomas and might therefore optimize the therapeutical strategy.

### 2.6
**Lymphoscintigraphy using double tracer technique in tumors of the head and neck**

S. Klutmann, S. Kröger, K.H. Bohuslavizki, S. Höft, J.A. Werner, W. Brenner, E. Henze, Depts. of Nuclear Medicine and ORL, Kiel, Germany

In the management of head and neck cancer preoperatively it is most desirable to predict lymph node involvement in order to identify aberrant lymphatic drainage. We report on a method of lymphoscintigraphy in double tracer technique.
Lymphoscintigraphy was performed pre-/intraoperatively in 78 patients. Patients received 100 Mbq Tc-99m-colloid in 3-4 peritumoral sites. 20 min later patients received 50 Mbq Tc-99m-pertechnetate i.v. for anatomical landmarking. Planar images were obtained at 30 min and 4-6 h from both anterior and lateral views using a LFOV-gamma camera.
28/78 = 36% of the patients showed no lymphatic drainage at all. Lymph node uptake could be assigned to the six known cervical lymphatic compartments in 50/78 = 64%. 36/78 = 46% showed unilateral, and 14/78 = 18% exhibited bilateral lymphatic drainage. Although in 13 out of these 14 patients the primary tumor was localized unilateral, lymph drainage was observed on both sides of the neck. In one of these patients the scintigraphic finding resulted in a more extended bilateral neck dissection.
In conclusion, lymphoscintigraphy in double tracer technique sufficiently visualizes lymphatic drainage in patients with head and neck cancer and, thus, effects surgical treatment planning.

### 3.1
**Diagnostic algorythm in neck masses**

M.S. Plouzhnikov, V.G. Merkoulov, Dept. of Otorhinolaryngology, University of St. Petersburgh, Russia

180 patients were assessed at pre-Clinic and post-op stages employing endoscopy, clinical investigation, X-ray, thermography, ultrasound scanning, CT, MRI, scintigraphy with tumourtropic drugs and laser correlation spectroscopy of the serum. Lymph nodes were examined with X-ray techniques as well as morphologically (fine-needle and surgical biopsies). Besides, post-op monitoring was carried out in 52 patilaryngeal cancer patients who underwent endoscopic laser surgery (32 patients) and extralaryngeal surgery with neck dissection. In 18 patients ultrasound scanning revealed lymphnodes enlargement and in 6 of them biopsy showed metastatic involvement. Comparative analysis of criteria accuracy demonstrated sensitivity of ultrasound scanning in 84.6% of cases, clinical examination was positive in 71.2% of cases whereas CT was positive in 73.4% out of 180 patients. However, CT was more beneficial in evaluating intimate relationships between deep lymph nodes and adjacent anatomical structures. Invasion into carotid arteries walls was easier discerned under MRI in the regimen of angiography or doppler scanning. Optical phenomenon of circulation immune complexes displacement towards 1000 A was found to be significant in clinically advanced laryngeal cancer.

### 3.2
**Diagnostic of nodal disease in the neck**

D. Bota, Dept. of Otolaryngology, University of Bucharest, Romania

The neck swellings are the most important disease in ENT medical practice in Romania. A great number of patients come to the doctor for tumours in the lateral regions of the neck. So, it is necessary to establish the etiology of the diseases, and knowing the frequences of them could be very useful. The incidence of clinically positive cervical neck nodes at the time of presentation varies both with the primary site and the patient population seen in our institution. The patients from lower socioeconomic strata seek medical attention when the neck swellings harm them.
**Material, patients and methods:** I had accomplished a study about diagnoses of nodal disease in the neck, using data obtained from reports of 231 patients, treated in our Clinic. Unknown neck mass workup protocol we had used in our clinic is:
1. History of the patient, 2. Physical examination, 3. Blood tests, 4. Endoscopy and biopsy (used in all patients suspected of having neoplasia), 5. CT-scan (to differentiate cysts from solid lesions; locates mass inside or outside a gland or a nodal chain, 6. Radionuclide scan (for thyroid lesion), 7. Arterigraphy (for vascular lesions), 8. Magnetic resonance imaging (useful for soft-tissue involvement by tumour), 9. Radiography; plain, 10. Skin tests (useful for diagnostic of chronic or granulomatous inflammatory lesion) 11. Needle biopsy (fine-needle aspiration biopsy), 12. Open biopsy, 13. Culture with sensitivity tests (for inflammatory tissue at open biopsy).
**Results:** In our lot of patients 79,65% had metastatic nodes, 8,55% inflammatory nodes and 9,91% other tumours of the neck. In all patients the primary lesion was unknown and the cervical swelling was the first symptome.
**Conclusion:** Because of high incidence of metastatic nodes in neck region, if the history, physical examination and routine diagnostic tests do not lead to a definitve diagnosis, any unknown neck mass, particularly a unilateral, asymptomatic mass corresponding to the location of known lymph node groups, must be considered a metastatic neoplastic lesion, until proved otherwise, it will be need supplimentary investigations especially for larynx.

3.3

**Cervical lymph node metastasis from squamous cell carcinoma of the upper aerodigestive tract**

B. Cizmarevic, A. Munda, V. Didanovic, T. Groseta, B. Drago, J. Rebol, C. France, Dept. of ORL, General Hospital Maribor, Slovenia

The status of the cervical lymph nodes is the single most important prognostic factor in the carcinoma of the upper aerodigestive tract. When cervical nodal metastases exist at presentation, cure rate dramatically decreases.

Cervical neck nodes were evaluated preoperatively and in histological specimens of 137 patients treated in our department between 1.1.1990 and 31.8.1994. Palpation and ultra sound were used for clinical assessment and so obtained preoperative N stage was compared with N stage obtained with histologic examination of operative neck dissection specimens. Clinical assessment in our patients was not accurate in spite of preoperative ultrasound investigations of the neck. In 29% of patients clinical N stage was higher than pathohistological one and in 11% of patients it was lower. This are results that could not satisfy us since all together 40% of necks were misjudged. We should think about finding a more accurate method of preoperative neck evaluation (fine needle biopsy?)

3.4

**Detection of nodal disease in patients with clinically N0 necks**

Z. Balatoni[1], K. Hrabák[2], J. Élo[1], Z. Kótai[1], [1]Dept. of Otorhinolaryngology and Head and Neck Surgery, Uzsoki District Hospital, [2]Transplantation Clinic, University of Budapest, Hungary

**Introduction:** Radiation therapy, modified or selective neck dissection are all appropriate options for the selective treatment of the neck when there is a high risk (20-30%) of occult metastasis, but methods unnecessarily increase the morbidity for patients with truly negative necks.

**Patients and methods:** In this prospective study we have examined those eligible patients who appeared at our clinic between June 1, 1995 and September 1, 1997 with oral, oropharyngeal and laryngeal cancers with clinically negative necks. Computed tomography was performed in 38 patients to image both the primary site and the neck. Every patient underwent surgical treatment with neck dissection. All neck dissection specimen were examined before fixation. The lymph nodes described by CT were dissected from the specimen. Routine hematoxylin-eosin staining and the histological examination of the neck contents were used to verify the findings of the CT. The criteria for cervical metastasis on CT included: nodes of any size with evidence of central necrosis; minimal axial diameter: 11 mm (subdigastric); 10 mm usually; grouping of 3 or more ill-defined nodes measuring 8-10 mm.

**Results:** 38 neck dissections were performed for clinically negative necks. The number of lymph nodes described by CT was 328. The histological findings have showed 25 metastatic and 303 negative nodes. There were 8 false negative and 2 false positive nodes. Sensitivity value of the CT-scan was 88.2%, specificity value was 95.8% concerning patients or necks. Considering all examined lymph nodes the sensitivity was 75.7% while the specificity was 99% of the CT.

**Conclusion:** We have detected 86.6% of the pN+ necks by the systematic use of CT in the pre-treatment assessment. We have concluded that CT significantly increased the certainty of the pre-operative diagnosis, allowing a better selection for neck dissection, and it made it possible to use a "wait and see" policy with the patients having a clinically and CT negative neck.

3.5

**Fine needle aspiration in lymph node metastasis of the head and neck**

E.Sprenger, Institute of Cytopathology, University of Kiel, Germany

The cytological diagnosis of palpable nodules in the head and neck comprises four organs, or tissues. These are the salivary glands, the thyroid and parathyreoid, the lymph nodes and the skin and soft tissues.

We shall focus here on metastases of malignant tumors in lymph nodes. In the differential diagnosis they need to be distinguished from both inflammatory lesions of the lymph nodes and malignant lymphomas

The most frequently seen metastases are those of squamous cell carcinoma of the oral cavity, pharynx, larynx and lung. The second most common are metastases of adenocarcinomas of the head and neck, the lung and the breast, less frequently also from the gastrointestinal tract, the pancreas and the prostate. Melanomas of the head and neck regions metastasize to the lymph nodes of these regions. Metastases of small-cell undifferentiated carcinomas are found especially in the supraclavicular lymph nodes. Now and then lymphoepithelial carcinomas of the nose and pharynx are only discovered because of their lymph node metastases.

The diagnosis of lymph node metastases of malignant tumors in the head and neck is recognized as a reliable, simple procedure that is tolerated well by the patient. Difficulties can arise in the diagnostic classification in cases in which the metastatic involvement of the lymph nodes is not total or in which there is extensive fibrosis or necrosis. In a small number of cases it can be difficult to differentiate metastases of malignant tumors from benign and malignant lymphoproliferative lesions. The problems are usually readily apparent and can than be solved histologically.

With its sensitivity of 98% and ist specificity of 99.7%, the cytological diagnosis of lymph node lesions is a reliable method that can be used in planning the further diagnostic and therapeutic procedures for metastasizing tumors of the head and neck.

3.6

**Sonomorphologic pattern of lymph node metastasis in head and neck cancer**

C. Arens, T. Klimek, C. Popella, H. Glanz, Dept. of Otorhinolaryngology, University of Gießen, Germany

**Background:** Ultrasound is an important diagnostic tool which is known as being very sensitive but not specific in diagnosis of head and neck metastasis. Therefore we tried to develop new sonomorphological criteria to detect lymph node metastasis in a preoperative stage.

**Material and methods:** Retrospectively we analyzed 200 ultrasound examinations of histologically proven neck metastasis of patients suffering from head and neck cancer.

**Results:** Not only lymph nodes over 2 cm in diameter but also spheroid like lymph nodes among 0.5 to 1.5 cm in diameter were found to have an accordance of 85% with the histologically proven positive lymph nodes. Spheroid lymph nodes show a low echogenity and a homogenous sonomorphological pattern.

**Conclusion:** The presented results suggest that it is possible to detect positive lymph nodes in head and neck cancer not only with a high degree of sensitivity but also specificity by using these criteria during a sonographic examination in a preoperative stage.

### 3.7

**B-scan sonography in the diagnostics of metastases of the neck**

C. Popella, C. Arens, T. Klimek, Dept. of Otorhinolaryngology, University of Gießen, Germany

**Background:** The diagnostics of lymph node metastases of the neck in patients with tumours of the head and neck includes sonography as an established method of examination. Beside palpatory evidence, sonography presents an inexpensive method for an experienced examiner and as a real-time procedure a sensitive method, equal to computertomography or even superior to it.
**Objective:** The question whether the probability of metastases is related to their size was examined by means of a comparison of the size of sonographically measured lymph nodes with histological results.
**Method:** Lymph nodes of 213 patients with tumours of the head and neck were categorized according to their size. After neck dissection, the specificity of sonography was checked by means of histological findings.
**Results:** Evidence of lymph nodes in sonography was positive in 84%, negative in 16%. Metastases were found in 55% of patients with sonographically detectable lymph nodes, nodes were enlarged unspecifically in 45%. Lymph nodes with a size of 0.5 - 1 cm showed metastatic growth in 27%, up to 1.5 cm in 31% and up to 2 cm in 58%. At a size of more than 2 cm, specificity lay by 89%. Depending on size and site of the primary tumour, the necessity of a neck dissection should be controlled with all patients who show sonographically detectable lymph nodes.

### 3.8

**The value of ultrasound examination in early diagnosis of nodal disease in the neck in follow up patients operated for head and neck cancer**

Z. Szmeja, M. Wierzbicka, J. Kaczmarek, M. Kordylewska, Dept. of Otolaryngology, University of Poznan, Poland

The purpose of this study was to evaluate the superiority of ultrasound examination of the neck in comparison with palpation in early detection of nodal recurrence in head and neck cancer patients. Close follow up with ultrasonography of patients after initial operation is essential to detect the recurrence early, while surgical salvage is still feasible. Ultrasound also proved to help in doing follow up and controls during radiation therapy. In this special group of patients nodal recurrence is difficult to detect early. Scars, oedema and anatomical changes due to prior surgery and radiotherapy make the clinical examination unreliable.
The group of 1257 patients (970 - larynx cancer, 149 - tongue and floor of mouth, 138 tonsil) operated between 1990-1996 were undergoing regular ultrasound examination (follow up). Nearly 6000 examination were performed in this group. 151 patients who developed nodal recurrence had surgical salvage; 47,6% of them (72 patients) had small, non-palpable lesions, 20% (31 patients) discreat and slight changes in the scarred neck. Neck re-operations succeed to be radical in 80,8%, in 122 patients; 85 patients (69%) who underwent successful salvage surgery had an early diagnosis with ultrasound imaging. The range of follow-up was 1-49 month. Comparison between patients with palpable lesions and detected sonographically was performed as regards: size of nodes, pattern of recurrence, possibility of performing radical surgery, the period of time from the last control examination to the moment of recurrence detection.

### 3.9

**Sonography for detection of late lymph node metastases in the head and neck region: an effective method of follow-up screening ?**

J. Quetz, M. Bosse, D. Sperlich, M.C. Heißenberg, Dept. of Otorhinolaryngology, Head and Neck Surgery, University of Kiel, Germany

**Purpose:** Sonography has proved more accurate than other methods in detecting cervical lymph nodes. But there is little knowledge about the efficiency in the follow-up after therapy of malignant tumors in the head and neck region. **Patients and methods:** From January 1989 to September 1997 10.372 sonographic examinations for detection of metastases were performed as a follow-up procedure. So far, 2.850 sonographic examinations in 427 patients were evaluated. The follow-up-period ranged from 18 to 80 months. Suspicious findings, according to sonomorphologic criteria, led to intensified diagnostics, e.g. shorter intervals, fine-needle aspiration cytology and operation. **Results:** Conspicuous lymph nodes were documented in 44% of the checks, in 15% of all examinations they were considered suspicious. In 4 patients, were palpation was negative, metastases actually were evident. In 24 cases, sonography was helpful in differentiating clinical findings. All cases are analysed and the benefit of 96 sono-guided cytologies is discussed. **Conclusion:** Sonomorphologic criteria for malignancy are less specific than expected. The screening-program was modified to only include patients with realistic options for further curative therapy. On-demand-sonography has proved to be reliable. A new documentation record for an easier and more precise documentation was designed.

### 3.10

**The value of ultrasound examination of lymph nodes of the neck in N0 patients with malignant tumours of the head and neck**

Z. Szmeja, J. Kaczmarek, M. Wierzbicka, M. Kordylewska, Dept. of Otolaryngology, University of Poznan, Poland

The analysis comprised 1257 patients operated in the Clinic of Otolaryngology University School of Medical Sciences in Poznan in the years 1991 - 1996. These patients were operated on malignant tumours of head and neck (970 larynx cancer, 149 tongue cancer, 138 malignant tumours of the tonsil).
All patients underwent palpation and ultrasound examination of the lymph nodes of the neck. In 554 patients (438 with larynx cancer, 72 with tongue cancer and 44 with malignant tumours of the tonsil) a palpation examination did not reveal enlarged lymph nodes. In this group of 554 patients (N0 in palpation examination) the ultrasound examination revealed the enlarged lymph nodes in 305 cases, from whom 234 patients had larynx cancer, 46 had tongue cancer and 25 with malignant tumours of the tonsil. The presence of histologically confirmed metastases in the surgically removed lymph nodes was found in 25,21% of patients with larynx cancer, 23,91% of patients with tongue cancer, and in 28% of patients with malignant tumours of the tonsil.

### 3.11

**Ultrasound and ultrasound-guided fine-needle aspiration cytology in the assessment of cervical lymph node status in head and neck cancer patients**

T. Atula[1], R. Grénman[1], M. Varpula[2], T. Kurki[2], P.-J. Klemi[3], [1]Dept. of ORL, [2]Imaging Center, [3]Dept. of Pathology, University of Turku, Finland

**Background:** Lymph node status of the neck is considered the most important tumor related prognostic factor in head and neck cancer patients. An accurate method for assessing the lymph node status may have therapeutic implication in these patients.
**Methods:** Altogether 86 consecutive head and neck cancer patients, who had no metastases in the neck on palpation, were examined using ultrasound (S) and ultrasound-guided fine-needle aspiration cytology (FNAC) to evaluate the additional information obtained by these methods.
**Results:** Among the 86 patients, altogether 63 FNACs were taken in 43 patients. FNAC taken under US-guidance showed malignancy in 13 (30%) patients (18 FNACs). The US size criteria used for malignancy were fulfilled in 7 of these patients, whereas the lymph nodes were of normal size in 6 of them. Some of these metastases could not be diagnosed with CT. FNAC showed multiple ipsilateral metastases in three patients and bilateral metastases in one patient.
**Conclusion:** US combined with US-guided FNAC can be recommended as a method for evaluation of regional metastases in head and neck cancer patients.

### 3.12

**The value of ultrasound with ultrasound-guided fine needle aspiration biopsy compared to computed tomography in the detection of regional metastases in the clinically negative neck**

R.P. Takes, P. Righi, C.A. Meeuwis, J.J. Manni, P. Knegt, R.J. Baatenburg de Jong et al., Leiden, Rotterdam, Nijmegen, The Netherlands, Indiana, USA

**Purpose:** The purpose of the present study was to compare the accuracy of ultrasound guided fine-needle aspiration biopsy (UGFGNAB) and computed tomography (CT) in detecting lymph node metastasis in the clinically negative neck.
**Materials and methods:** Sixty-four neck sides of patients with head and neck squamous cell carcinoma (HNSCC) were examined preoperatively by UGFNAB and CT at one of five participating medical centers. The findings were correlated with the results of histopathologic examinations of the neck specimen.
**Results:** UGFNAB was characterized by a sensitivity of 48%, specificity of 100%, and overall accuracy of 79%. Three cases had non-diagnostic aspirations using UGFNAB and were excluded. CT demonstrated a sensitivity of 54%, specificity of 92% and overall accuracy of 77%. UGFNAB detected two additional metastases not visualized on CT whereas CT did not detect any metastases not seen on UGFNAB. The results of UGFNAB were similar between the participating centers.
**Conclusions:** Approximately one-half of the clinically occult nodal metastases in our patient group were identified by both CT and UGFNAB. Overall, UGFNAB and CT demonstrated comparable accuracy. The sensitivity of CT was slightly better than UGFNAB but the latter remains characterized by a superior specificity. The results of CT and UGFNAB did not appear to be supplementary.

### 3.13

**Diagnostic accuracy of pretherapeutic lymph node ultrasonography in patients with oral cancer - a histologically controlled study**

H. Terheyden[1], B. Fleiner[1], J. Lüttges[2], [1]Dept. of Oral and Maxillofacial Surgery, [2]Inst. of Pathology, University of Kiel, Germany

**Aim of the study:** The concept of prophylactic complete neck dissection in patients with oral squamous cell carcinoma (SCC) > T1 leads to overtreatment and many cases of histologically negative neck dissection specimens. Selective neck dissection of lymph node (LN) compartments based on pretherapeutic sonography would be an alternative. The aim of this study was to evaluate the accuracy of LN sonography in a histologically controlled study.
**Materials and methods:** 30 patients with oral SCC (T2 - T4) underwent bilateral preoperative sonography (B-scan, 7.5MHz linear scanner). The neck dissection specimen was anatomically marked in 4 levels (ADT classification). After fixation the specimen was cut in 2mm slices (lamellation). If LN were present, serial histologic sections (hemotoxilin eosin) were performed.
**Results:** In a total of 469 histologically identified LN 428 had previously been detected sonographically. Among 30 metastases (prevalence 6,4%) 29 had been identified sonographically and one had been missed (level I, 5mm diameter). The sensitivity was 96.7%, specificity 9.5%. The cut off level for LN detection was >5mm in level I-IV. The mean size in mm of non detected LN was 6.8 (I), 5.9 (II), 4.9 (III), 4.8 (IV) indicating that accuracy improved with the level.
**Conclusion:** Due to inconstant sonomorphologic criteria for malignancy a sonografically detected LN has to be classified as putative metastasis. In this study the sensitivity for detection of metastases was high at a low specificity. From a diagnostic viewpoint the safeness of sonographically based selective neck dissection would be satisfactory. Combination with ultrasonographic fine needle aspiration biopsy may improve the specificity.

### 3.15

**Prognostic significance of colour doppler findings in combination with CT-scan in cervical lymph node metastases of head and neck tumors**

M. Helbig[1], A. Dietz[1], S. Delorme[3], I. Zuna[3], V. Rudat[2], C. Conradt[4], H. Weidauer[1], [1]Dept. of ORL, [2]Dept. of Rad. Oncology, [4]Inst. of Medical Biometry, University of Heidelberg [3]German Cancer Research Centre, Heidelberg, Germany

**Introduction:** Cervical lymph node metastases in 25 patients with head and neck cancer were examined before combined, accelerated radiochemotherapy by doppler sonography and CT-scan.
**Patients and Methods:** The findings were quantified using a computer-assisted protocol that quantitatively describes colour Doppler images by the relative colour pixel density (CPD). Tumor volume (including primary tumor and neck nodes) was quantified by using a computer-analysis of tumormargins in CT-Scans.
**Results:** In the patient group with a CPD below median, the median survival was 958 days, in the group with a higher CPD it was 423 days (p=0.05). The time to detection of distant metastases was 18 months with a low CPD, and 6 months with a high CPD (p=0.05). In addition of tumorvolume, the patients with CPD and tumorvolume below median showed a significant better survival in comparison to the patients with higher tumorvolume and higher CPD (p=0.0015).
**Conclusions:** In patients with metastatic head and neck cancer, highly vascularised lymph nodes detected with colour Doppler sonography and high tumorvolume may indicate a shorter survival and earlier occurrence of distant metastases.

**3.16**

**Detection of lymph node metastases in head and neck carcinoma: sensitivity and specificity of MR-angiography**

F. Donnerstag[1], R. Knecht[2], A. Möller-Hartmann[3], F. Zanella[3],
[1]University of Bochum, [2,3]University of Franfurt, Germany

**Purpose:** To find out the capability of MR-angiography (MRA) in detection of metastatic spread in cervical lymph nodes.

**Material and methods:** Retrospectively the appearance of lymph nodes in 12 patients with head and neck carcinoma obtained by MR-angiography was correlated with the histopathological specimen of lymph nodes after neck dissection. Standardized Maximal Intensity Projections of MRA was reviewed independently by 2 radiologists. Only the detection of a avascular zone in hypervascularized lymph nodes was charged as metastatic involvement, the appearance of inhomogeneous lymph node vascularization was not drawn in account. 118 histopathological lymph node specimens were correlated with 75 radiological detected lymph nodes.

**Results:** The specifity of the avascular zone in hypervascularized lymph nodes was 100%, sensitivity was 38% only.

**Conclusions:** Sensitivity of the appearance of the avascular zone in lymph nodes is too low for detection of lymph node metastases. Evaluation of the inhomogeneous vascularized lymph node may be subject of further investigations.

### 2.7

**FDG - PET compared to CT and MRI in the staging of lymph node metastasis**

H. E. Eckel[1], M. Jungehülsing[1], U. Pietrzyk[3], K. Scheidhauer[2], [1]Dept. of ORL, [2]Dept. of Nuclear Medicine, University of Cologne, [3]Max Planck Inst. for Neurologic Research, Cologne, Germany

**Introduction:** In squamous cell carcinomas of the head and neck (SCCHN) staging is essential for the choice of the appropriate therapy. Especially the lymph node involvement has been shown to be an important prognostic factor, and therapy and surgical approach depend strongly on the findings of head and neck cross-sectional imaging techniques as computed tomography and magnetic resonance imaging. Their detailed morphologic informations usually are sufficient for TNM-staging. Nevertheless in patients with lymph nodes sized 1 cm or in patients with large SCCHN without proven metastatic lymph node spread the conventional imaging techniques are not always satisfying. 2-Desoxy-$^{18}$Fluoro-D-Glucose positron emission tomography (FDG - PET) has been shown to be a sensitive metabolic marker for the detection of metastatic spread.

**Purpose:** To compare the sensitivity of FDG-PET and the above-mentioned cross-sectional imaging techniques, 25 patients suffering from SCCHN (T2N0M0 - T4N3Mx) underwent computed tomography, magnetic resonance imaging and FDG-PET. Evaluation was performed using the histologic findings of neck dissection.

**Results:** In 6/25 patients only FDG-PET showed additional suspect lymph nodes and contralateral / bilateral neck dissection was performed. In 4/6 patients these lymph nodes showed metastatic infiltration histologically In 1 patient an unknown lung metastasis was detected by FDG-PET.

We think FDG-PET should be performed routinely in patients with uncertain lymphatic metastatic spread and possible consequences for the choice of therapy.

### 2.8

**$^{18}$ FDG PET is a sensitive tool for the detection of the occult primary cancer (CUP-syndrome)**

M. Jungehülsing[1], K. Scheidhauer[2], U. Schroeder[1], E. Voth[2], H. Eckel[1], U. Pietrzyk[3], H. Schicha[2], [1]Dept. of ORL, [2]Dept. of Nuclear Medicine, University of Cologne, [3] Max Planck Inst. for Neurologic Research, Cologne, Germany

**Introduction:** The head and neck lymph nodes are the most frequent manifestation site of carcinomas of unknown primary (CUP syndrome). 18-Fluordeoxyglucose - Positron Emission Tomography (18-FDG-PET) has been shown to be a very sensitive tool for detecting primary malignant lesions as well as metastatic spread.

**Purpose:** We have prospectively investigated the sensitivity of 18-FDG-PET in detecting occult primary carcinomas with head and neck lymph node metastases.

**Patients and methods:** From 5/94 until 8/97, in 477 patients malignant lesions were diagnosed at the University of Cologne ENT outpatient clinic. The routinely performed staging procedures were endoscopy of the pharynx, the larynx and the esophagus, computed tomography resp. magnetic resonance imaging of the skull base, neck and thorax , laboratory analyses including EBV-AVC serology, and bone scintigraphy. After the staging in 16 / 477 patients (3,3 % ) a CUP syndrome had to be presumed the primary cancer not being detected. In these patients 18-FDG-PET was performed on a Siemens Ecat Exact 921 PET Scan with 370 MBq $^{18}$FDG ; images were reconstructed using a transmission - emission fusion technique.

**Results:** In 7/16 patients (44%) 18-FDG-PET revealed an unknown primary: in 3 patients a bronchial carcinoma, in 2 patients a nasopharyngeal carcinoma, in 1 patient a squamous cell carcinoma (SQCC) of the parotid gland and in 1 patient a SQCC of the base of the tongue. In one patient an adenocarcinoma of the lung was found showing a histology completely different to the SQCC lymph node metastasis, so that a second occult tumor had to be presumed. 18-FDG-PET made possible surgery in 5/16 patients in curative intention. We think the 18-FDG-PET is necessarily to be performed in all patients suffering from a stringent CUP syndrome.

### 3.14

**Duplex echographic lymph node vascularisation - prognostic indicator for lymph node involvement in head and neck cancer**

M. Westhofen, Dept. of Otorhinolaryngology, University of Aachen, Germany

During the last decade echography has become part of the head and neck diagnostic procedures. Many investigations were presented to demonstrate the sensitvity and specifity of the echographic findings. Now the duplex echography of head and neck tumours and lymph nodes can provide image analysis of tumour and lymph node vascularisation in scan images.

For this purpose contrast enhancers can be administered intravenously to see even smallest intranodal vessels with low blood flow of <5cm/sec. The overlay of high resolution gray scale echography and colour coded duplex echography allows imaging of the lymph node´s substructure including hilus, paracortical area and tumour destruction and compression of intranodal vessels. During the last years, typical vascularisation patterns for squamous cell carcinoma of the neck and lymph node metastases of these tumours as well as thyroid and kidney cancers were characterised. Meanwhile, indication and contraindication for surgical treatment is based upon duplex echographic findings. Duplex echography is routinely performed by the head and neck surgeon himself to support the preoperative planning with echographic imaging adopted to the site of tumour. To hold high standards of imaging quality control rules for the examination and criteria for the image documentation were elaborated. During the last year, the acceptance of echographic images was improved by use of a new topographic imaging procedure (Siescape, Siemens, Erlangen, Germany). By use of this procedure scans of the whole neck can be demonstrated in any scan direction and without limitation of the scan size. Selective partial images can be picked out for any detailed investigation after having performed the scan. So, the size of the scan is similar to the radiologic scan techniques, whilest the resolution and flexibility is due the echographic criteria. The experience of the last 12 months shows an increase of early tumour recurrence diagnosis by use of echographic technique prior to other diagnostic procedures in 7 % of primary tumour incidence. Reorganisation of postoperative care and follow-up and intense integration of the new echogrpahic methods will improve the chance of surgical recurrence treatment.